# Immune-related adverse events of biological immunotherapies used in COVID-19

**DOI:** 10.3389/fphar.2022.973246

**Published:** 2022-08-25

**Authors:** Daniela Baracaldo-Santamaría, Giovanna María Barros-Arias, Felipe Hernández-Guerrero, Alejandra De-La-Torre, Carlos-Alberto Calderon-Ospina

**Affiliations:** ^1^ Pharmacology Unit, Department of Biomedical Sciences, School of Medicine and Health Sciences, Universidad del Rosario Bogotá, Bogotá, Colombia; ^2^ Neuroscience Research Group (NEUROS). Neurovitae Center, Escuela de Medicina y Ciencias de la Salud, Universidad del Rosario, Bogotá, Colombia; ^3^ Center for Research in Genetics and Genomics (CIGGUR), Escuela de Medicina y Ciencias de la Salud, Universidad del Rosario, Bogotá, Colombia

**Keywords:** COVID-19, monoclonal abs, tocilizumab, sarilumab, siltuximab, sotrovimab, adverse reactions

## Abstract

The use of biological immunotherapeutic drugs is one of the options currently being evaluated and employed to manage COVID-19, specifically monoclonal antibodies, which have shown benefit by regulating the excessive immune response seen in patients with severe infection, known as a cytokine storm. Tocilizumab has received particular importance for this clinical application, as has sarilumab. Both drugs share a substantial similarity in terms of pharmacodynamics, being inhibitors of the interleukin six receptor (IL-6Rα). Furthermore, sotrovimab, a neutralizing anti-SARS CoV-2 antibody, has gained the attention of the scientific community since it has recently been authorized under certain circumstances, positioning itself as a new therapeutic alternative in development. However, despite their clinical benefit, biological immunotherapies have the potential to generate life-threatening immune-related adverse events. Therefore it is essential to review their incidence, mechanism, and risk factors. This review aims to provide a comprehensive understanding of the safety of the biological immunotherapeutic drugs currently recommended for the treatment of COVID-19, provide a review of the known immune-mediated adverse events and explore the potential immune-related mechanisms of other adverse reactions.

## 1 Introduction

The COVID-19 pandemic caused by the severe acute respiratory syndrome coronavirus 2 (SARS-CoV-2) has, as of 06 March 2022, led to a reported ∼6 million deaths worldwide (World Health Organization, 2022). With the emergence of the pandemic in early 2020, many drugs were hypothesized to have a potentially beneficial role in the pathophysiology of the disease, including antiparasitic, antibiotic, antiviral, and even disinfectant substances (Caly et al., 2020; Fantini et al., 2020; Kang et al., 2020). However, 2 years later, it has been confirmed that many of the initially proposed drugs don’t have efficacy against COVID-19 (hydroxychloroquine, chloroquine, lopinavir, ritonavir, azithromycin, ivermectin) and, actually, the infodemic led to an increase in the incidence of adverse drug effects and self-medication, contributing to drug misuse (Repurposed Antiviral Drugs for Covid-19 — Interim WHO Solidarity Trial Results, 2021; The Recovery Collaborative group, 2020). Despite the remarkable efficacy of novel vaccines, therapeutics continue to be studied and developed due to the threat of emerging variants and the waning of vaccine-induced protection (Piechotta and Harder, 2022).

With a further understanding of the pathophysiology of COVID-19, the disease was described to evolve in overlapping phases (Griffin et al., 2021). First, the viral stage is asymptomatic or mild in most patients; then, the disease may progress to a severe or critical state in some patients. Patients with COVID-19 that have developed a severe disease often have a phase characterized by hyperresponsiveness of the immune system. Numerous studies have shown increased levels of IL-6 (interleukin 6), IL-1 (interleukin 1), and TNF-α (Tumor Necrosis Factor-alpha) (Schroeder and Bieneman, 2022) that correlate with disease severity (del Valle et al., 2020a; Zhu et al., 2020; Chen et al., 2020). The documented presence of significantly elevated plasma levels of cytokines led to the evolving concept of COVID-19-induced cytokine storm syndrome (CSS). CSS is characterized by overwhelming systemic inflammation, organ dysfunction, hemodynamic instability and, in some cases, death. This syndrome is not a disease itself, but rather, it is the expected outcome of diverse insults such as autoimmune diseases and infections (Fajgenbaum and June 2020a; Rodríguez et al., 2020).

Clinically CSS can manifest as an influenza like-syndrome that can lead to multi-organ failure. Fever is the most encountered symptom, and the most severe cases often present with higher temperatures (Fajgenbaum and June 2020b). Patients may also present with fatigue, anorexia, arthralgia, myalgia, rash and diarrhea. These symptoms may be attributed to direct cytokine-induced tissue damage or to immune-cell-mediated responses. CSS can lead to disseminated intravascular coagulation, dyspnea, acute kidney injury, liver damage, hypoxemia, stress related cardiomyopathy, encephalopathy and capillary leak syndrome. Laboratory findings in patients with CSS include blood count abnormalities like leukopenia, leukocytosis, anemia, thrombocytopenia, elevated levels of C reactive protein, D dimer, ferritin, and triglycerides, and hypoalbuminemia (Zanza et al., 2022).Serum concentrations of cytokines in COVID-19 patients have been correlated with disease severity and shorter survival (del Valle et al., 2020b). Consequently, the role of immune dysregulation and CSS in COVID-19 has prompt the investigation of multiple immunomodulatory drugs (Eynde et al., 2022).

Knowing that the immune response to the pathogen, rather than the pathogen itself, may contribute to severe disease and organ failure, immunomodulators were introduced as a potential therapy for COVID-19 (Immunomodulators, 2022; McCarthy, 2022). The two classes of IL-6 inhibitors, the monoclonal antibodies against the IL-6 receptor (sarilumab, tocilizumab) and the anti-IL-6 monoclonal antibodies (siltuximab), have been studied in various clinical trials, systematic reviews, and meta-analyses. Some have been shown to reduce the need for mechanical ventilation and mortality rates in COVID-19 patients (Rubio-Rivas et al., 2021; Gupta et al., 2022a; Moosazadeh and Mousavi, 2022). While biological immunotherapies have improved outcomes in COVID-19 patients, they may stimulate innate and adaptive immune responses, causing an immune reaction to the drug, known as an immune-related adverse event (irAE). Safety concerns regarding irAEs have become of interest given the severity of some of these adverse reactions and the increased off-label use of many biological compounds because of the pandemic. Understanding the molecular mechanisms behind them is essential to guide physicians towards proper prevention, diagnosis, and treatment, while prioritizing monitoring.

This review aims to provide a comprehensive understanding of the safety of biological immunotherapeutic drugs used for the treatment of COVID-19, with particular emphasis on the most frequent and severe immune-related adverse reactions. In addition, we will clarify the molecular mechanisms by which they are produced and evaluate if some type B reactions to these biological immunotherapies could have an immune-related mechanism. The drugs included in this revision correspond only to those currently recommended for treatment of COVID-19.

## 2 IL-6 pathway inhibitors

### 2.1 Tocilizumab

Tocilizumab is a recombinant humanized monoclonal antibody that binds to circulating and membrane-expressed IL-6 receptors, thereby competitively inhibiting the binding of IL-6 to its receptor. The FDA approved it to treat several autoimmune and inflammatory conditions, and it is most frequently used in the treatment of rheumatoid arthritis (Actemera, 2022). Given the association linking critical and fatal COVID-19 with increased pro-inflammatory cytokines, including IL-6, tocilizumab has been approved by some regulatory agencies to treat COVID-19 in adults receiving systemic corticosteroids, supplemental oxygen, or who are under mechanical ventilation (Actemra/RoActemra, 2022). Tocilizumab has been studied in numerous randomized controlled clinical trials (RCTs) in hospitalized patients with COVID-19. An updated meta-analysis regarding tocilizumab efficacy in COVID-19 patients found that tocilizumab significantly decreased the 28–30-days mortality and the incidence of mechanical ventilation and intensive care unit admission (Zhang et al., 2022). In addition, some studies included in this meta-analysis showed a significant difference between the tocilizumab-treated group and the control group in the number of reported serious adverse effects (Stone et al., 2020; Hermine et al., 2021; Rosas et al., 2021; Soin et al., 2021).

#### 2.1.1 Safety

Generally, tocilizumab has a good safety profile; however, relevant adverse reactions have been reported. Rosas et al. reported adverse events in 77.3% of 295 patients, of which 34.9% were serious adverse events. The most frequently reported adverse drug reactions were infection, hypersensitivity, abnormal liver function tests, myocardial infarction, and bleeding, among others (Rosas et al., 2021). Furthermore, in some clinical trials, the tocilizumab-treated arm also had more serious adverse events than the standard care group. The most frequent were respiratory distress syndrome, shock, and cardiac disorders (acute coronary syndrome, arrhythmias, bradycardia, myocarditis) (Soin et al., 2021). The highest risk for secondary infection has also been documented with tocilizumab (Theinvestigators, 2021; Boppana et al., 2022). Other adverse reactions reported using tocilizumab have been anaphylactic reaction, anaphylactic shock, renal failure, pulmonary fibrosis, drug-induced liver injury, pancreatitis, and pancytopenia (Gatti et al., 2021). Other adverse events described on the product label include infusion reactions (angioedema, rash, etc.). The most serious adverse drug reactions are severe infection and hypersensitivity reactions.

#### 2.1.2 Immune-related adverse drug reactions

##### 2.1.2.1 Anaphylactic and hypersensitivity reactions

Clinical data on anaphylactic reaction to tocilizumab during the treatment of COVID-19 is limited. However, extrapolated information from the treatment of rheumatic diseases has shown that anaphylaxis and other hypersensitivity reactions that require treatment discontinuation occur in approximately 0.1% of patients receiving intravenous tocilizumab (Park et al., 2019; Actemera, 2022). In addition, there are case reports of tocilizumab-induced anaphylaxis in patients with COVID-19, where tocilizumab was given as an infusion of 8 mg/kg every 12 h. Patients developed anaphylactic shock after the first or second infusion, preceded by pruritus and shortness of breath, suggesting an immunoglobulin E (IgE)-mediated mechanism and prior sensitization to tocilizumab (Atayik and Aytekin, 2021). Delayed hypersensitivity reactions have also been documented; several cutaneous adverse events like erythroderma, cutaneous vasculitis, and psoriasiform rash have been reported (Yoshiki et al., 2010; Palmou-Fontana et al., 2014; Sakaue et al., 2014; Wu et al., 2015) with skin biopsies that show CD4^+^T cells and eosinophil infiltration in the upper dermis. Infusion-related hypersensitivity reactions, from life-threatening reactions like angioedema to less severe reactions like rash, urticaria, epigastric discomfort, and headache, are also described in the literature (Actemra/RoActemra, 2022). Skin tests (skin prick tests or intradermal tests) have been used to detect hypersensitivity reactions at a non-irritant concentration of 20 mg/ml, where a wheal area of ≈ 3 mm^2^ is considered positive (Rocchi et al., 2014). In addition, successful cases of desensitization to tocilizumab have been reported with small weekly doses and premedication (Cansever et al., 2018; Justet et al., 2014). Risk factors for the development of hypersensitivity reactions to tocilizumab have not been widely described; however, Yasuoka et al. documented that patients with sJIA (systemic juvenile idiopathic arthritis) that had light weight, younger age, and increased disease severity may have an increased risk of developing a hypersensitivity reaction (Yasuoka et al., 2019).

##### 2.1.2.2 Hematological abnormalities

Neutropenia is a relatively common adverse reaction to the use of tocilizumab, and it has been described in multiple studies in rheumatic diseases (Nakamura et al., 2009; Shovman et al., 2014; Moots et al., 2017). Tocilizumab-induced neutropenia in COVID-19 has also been documented in a study that evaluated adverse drug events reported in VigiBase (maintained by the World Health Organization). A total of 1,005 adverse drug events were found, including neutropenia, hypercoagulable states, anemia, cardiac arrest, atrial fibrillation, intestinal perforation, ulcers, hepatitis, hypersensitivity, reactivation of latent infections, among others (Charan et al., 2021). Neutropenia was the most frequently reported adverse drug event in the category of blood and lymphatic system disorders, occurring in 1.5% of the patients (513), followed by hypercoagulable state/hypofibrinogemia (0.8%) and anemia (0.5%) (Charan et al., 2021).

The exact mechanism by which tocilizumab produces neutropenia is not clear. However, based on *in vitro* evidence (Suwa et al., 2000) it is thought that IL-6 induces the release of neutrophils from marginated pools in the bone marrow; thus, inhibition of the IL-6 cascade might reverse this mechanism and cause neutropenia (Moots et al., 2017). Tocilizumab-induced neutropenia is dose-dependent, according to data from clinical trials in rheumatic diseases (Kremer et al., 2011; Benedetti et al., 2014). Neutropenia can be classified according to the absolute neutrophil count as mild (1.0–1.5  ×  10^9^/L), moderate (0.5–1.0  ×  10^9^/L), or severe (<0.5  ×  10^9^/L) (Boxer, 2012). Therefore, tocilizumab is currently not recommended in COVID-19 patients with an absolute neutrophil count of <1,000 per mm^3^ or a platelet count below 50,000 per mm^3^ (Fact Sheet For Healthcare Providers, 2021).

Prolonged and severe episodes of neutropenia during treatment with tocilizumab can cause an increased risk of developing serious infections. In COVID-19, the risk of infection has been documented principally in the following RCTs: the EMPACTA (NCT04372186) clinical trial showed that serious infections occurred in 5.2% of the tocilizumab-treated arm vs. 7.1% in the placebo group, with urinary tract infection being the most frequent disease and more commonly observed than in the placebo group (Salama et al., 2020); in the REMDACTA (NCT04409262) clinical trial, pneumonia was the most common infection reported and occurred more frequently than in the placebo group (Hoffmann-La Roche, 2022); information extrapolated from studies of tocilizumab in rheumatic diseases shows that infections reported after treatment are active tuberculosis, cellulitis, herpes zoster, gastroenteritis, diverticulitis, sepsis, bacterial arthritis, and invasive pulmonary infections that include candidiasis, aspergillosis, coccidioidomycosis, and pneumonia by *Pneumocystis jirovecii* (CHMP, 2021).

Tocilizumab-induced thrombocytopenia is reported on the product label, and it is a known possible adverse reaction to tocilizumab treatment. In patients with rheumatoid arthritis, thrombocytopenia has been reported in 8–9% of treated patients (Gabay et al., 2013). In COVID-19, there have been reports in RCTs of tocilizumab-induced thrombocytopenia. In the COVACTA trial (NCT04320615) thrombocytopenia was reported in 4% (n = 11) of patients in the treatment arm vs. 1% (n = 2) in the placebo group (HoffmannRoche, 2022). In the REMDACTA trial, thrombocytopenia was documented in 3% (n = 14) of the patients receiving tocilizumab (Hoffmann-La Roche, 2022). As with neutropenia, the exact mechanism by which tocilizumab induces thrombocytopenia is not clearly elucidated, but the hypothesis points to the role of IL-6 in increasing platelet levels during inflammation. Some studies have documented that IL-6 may enhance thrombopoietin mRNA transcription (Kaser et al., 2001). This suggests that tocilizumab would inhibit the IL-6-induced thrombopoietin expression and thus results in thrombocytopenia. To the best of our knowledge, no immune mechanism has been described for tocilizumab-induced hematologic abnormalities. They seem to be related to the drug’s mechanism of action (type A reaction).

##### 2.1.2.3 Interstitial lung disease

Drug-induced interstitial lung disease (DILD) is an infrequent adverse effect caused by tocilizumab; nevertheless, there have been post-marketing reports in large retrospective cohort studies (Curtis et al., 2015) and RCTs (Smolen et al., 2008; Fact Sheet For Healthcare Providers, 2021). The principally reported DILDs are acute pneumonitis, idiopathic pulmonary fibrosis, and exacerbation of rheumatoid arthritis-associated interstitial lung disease (Kawashiri et al., 2012; Sangüesa Gómez et al., 2016; Gouveia et al., 2020; Silva et al., 2020; Sugihara et al., 2021). The mechanism whereby tocilizumab induces interstitial lung disease is unknown; however, two mechanisms for DILD are commonly described. The first is cytotoxic lung injury, where the drug causes direct damage to lung tissue in a dose-dependent manner (Schwaiblmair et al., 2012). It is unlikely for tocilizumab to have a direct cytotoxic effect on lung tissue and, as far as we know there are no studies documenting this. This suggests that the possible mechanism for tocilizumab-induced interstitial lung disease may be immune-mediated. Immune-mediated DILD is not fully understood; what is known is that drugs can act as potential antigens or haptens, triggering an immune response that could potentially lead to immune-mediated lung toxicity. Another plausible mechanism is the generation of anti-drug antibodies that could lead to the formation of antigen-antibody complex deposition (Table 1), leading to pulmonary edema and interstitial lung disease (Sigaux et al., 2017).

**TABLE 1:** Mechanisms of irAEs with the use of tocilizumab.

Drug	irAEs	Mechanism	Comments	Reference
Tocilizumab	Hypersensitivity reactions: anaphylaxis and cutaneous AEs	Anaphylaxis: IgE-mediated hypersensitivity reaction	Younger age, lightweight, and increased disease severity (of sJIA) are risk factors that may be associated with an increased risk of developing a hypersensitivity reaction	Yasuoka et al. (2019) ^a^
		Cutaneous AEs: Delayed hypersensitivity reaction		
	Drug-induced interstitial lung disease	Immune complex-mediated reaction?	-	Schwaiblmair et al. (2012)
	Immunogenicity	Anti-tocilizumab antibodies	Very rare, but it can induce anaphylaxis or loss of efficacy	Sigaux et al. (2017)

sJIA, systemic juvenile idiopathic arthritis.

a The risk factors are described for the development of hypersensitivity reactions in patients with sJIA Yasuoka et al., (2019).

##### 2.1.2.4 Hepatic adverse events

Liver enzyme abnormalities have been frequently reported with the use of tocilizumab in rheumatic diseases and COVID-19. Studies on COVID-19 have documented that approximately 5–29% of patients treated with tocilizumab show elevated hepatic enzyme levels (Campochiaro et al., 2020; Morena et al., 2020; Charan et al., 2021). Hepatitis, hepatic cirrhosis, acute hepatic failure, hepatic steatosis, ischemic hepatitis, and worsening of autoimmune hepatitis have been reported in a pooled analysis of a long-term clinical trial in rheumatoid arthritis (Genovese et al., 2017). The mechanism of Tocilizumab-induced liver injury is not clearly elucidated, but there are many probable mechanisms. First, there seems to be a relationship linking IL-6 signaling with regulation of liver regeneration and vulnerability to injury. IL-6 is also thought to prime hepatocytes to re-enter the cell cycle from G_0_. In the absence of IL-6, liver regeneration is impaired, and it is more vulnerable to injury. For instance, IL-6 deficient mice are more susceptible to liver injury, showing increased hepatocellular injury and defective regeneration following treatment with carbon tetrachloride (used to simulate drug-induced liver injury) (Kovalovich et al., 2000). Secondly, there are case reports with biopsies that demonstrate focal necrosis of hepatocytes, steatosis, and early fibrosis, suggesting a direct mechanism of drug toxicity (Mahamid et al., 2011). However, no immune-related mechanisms have been described for the observed hepatic injury.

##### 2.1.2.5 Immunogenicity

The development of tocilizumab-specific anti-drug antibodies is rare, but it has been documented in various studies. For example, Ogata et al. found that 3.5% of the patients analyzed tested positive for anti-tocilizumab antibodies in a phase III study evaluating the efficacy and safety of tocilizumab in rheumatoid arthritis. However, no severe hypersensitivity reactions were reported in these patients, nor a lack of efficacy (Ogata et al., 2014). Sigaux et al. showed similar results, only finding 3.3% (n = 3) of patients with positive titers of anti-tocilizumab antibodies (Sigaux et al., 2017). This illustrates that tocilizumab triggers an immune response in some patients and could be the mechanism behind some adverse drug reactions, which has not been clarified. Although the results in these studies did not show severe hypersensitivity reactions in the patients with anti-tocilizumab antibodies, there are reports of serious adverse events of urticaria and angioedema.

#### 2.1.3 Recommendations

The monitoring recommendations available for tocilizumab when used in COVID-19 patients include measuring liver enzymes before starting the treatment. In patients presenting symptoms of liver injury, tocilizumab should be discontinued if ALT (alanine transaminase) or AST (aspartate aminotransferase) are greater than 5x ULN (upper limit of normal). In addition, neutrophil and platelet counts should be monitored as they have been associated with neutropenia and thrombocytopenia (Actemera, 2022). Neutrophils and platelets should be measured before treatment, 4–8 weeks after therapy initiation, and every 3 months. Tocilizumab should not be administered during the course of other active infections, and caution is advised in patients at risk of gastrointestinal perforation.

### 2.2 Sarilumab

Sarilumab is a human recombinant IgG1 antibody that, like tocilizumab, binds to IL-6 receptors and inhibits IL-6 signaling cascade. Its use is approved for treating rheumatoid arthritis, but it has been evaluated as a potential treatment for COVID-19. The efficacy of sarilumab in COVID-19 is controversial: some RCTs show better outcomes with early IL-6 blockade through a single dose of sarilumab (Merchante et al., 2022), while others have failed to show significant improvements in clinical status or mortality (Sivapalasingam et al., 2022). However, sarilumab is recommended by some guidelines as an alternative to tocilizumab in the treatment of COVID-19 (Nih, 2022). Sarilumab is recommended by the intravenous route, in addition to corticosteroids, in hospitalized patients with elevated markers of inflammation who require high flow oxygen supplementation or noninvasive mechanical ventilation. It is also recommended for ICU patients within 24 h of admission who require mechanical ventilation (Nih, 2022).

#### 2.2.1 Safety

Safety outcomes in some RCTs have shown that sarilumab-treated patients had more secondary respiratory bacterial infections, respiratory failure, and neutropenia (García-Vicuña et al., 2022). Another RCT evaluating the safety profile of sarilumab in COVID-19 patients reported that more patients in the treatment arm had elevations in liver function tests compared to the placebo group (Sivapalasingam et al., 2022). Similar studies have reported an increased incidence in the number of serious bacterial and fungal infections (Hermine et al., 2021). Long-term safety analysis extrapolated from phase III clinical trials of sarilumab use in rheumatoid arthritis shows that the most frequent adverse events were neutropenia, increased alanine aminotransferase, erythema at the injection site, upper respiratory tract infections, urinary tract infections, and bronchitis (Genovese et al., 2020).

#### 2.2.2 Immune-related adverse drug reactions

##### 2.2.2.1 Hypersensitivity reactions and immunogenicity

The development of anti-drug antibodies against sarilumab has been reported, with an incidence of 12.3% in an open-label clinical trial (NCT02121210) that evaluated the immunogenicity of sarilumab monotherapy in patients with rheumatoid arthritis. Of the reported patients with anti-drug antibodies, 10.8% had transient neutralizing antibodies (Wells et al., 2019). Adverse events reported in this study included a few hypersensitivity reactions. However, there were no notable differences in hypersensitivity reactions between the anti-drug-antibody-positive patients and the negative patients. Having a positive anti-drug antibody test did not have an impact on efficacy (Wells et al., 2019), in accordance with other studies (Tanaka et al., 2021). Even though sarilumab is a fully human monoclonal antibody, it still has the potential to generate an immune response, primarily because of unique sequences in the antigen-binding domain or because of post-translational modifications (Harding et al., 2010). Other reported hypersensitivity reactions are injection site rash and urticaria (Food and Drug Administration, 2022a). Desensitization protocols have not been proposed.

##### 2.2.2.2 Hematological abnormalities

Neutropenia is the most commonly reported hematological abnormality with the use of sarilumab. Absolute neutrophil counts of less than 1,000 per mm^3^ are reported in approximately 6% of treated patients with rheumatic diseases. However, an association has not been found between sarilumab-induced neutropenia and the increased risk of infection (Fleischmann et al., 2020; Food and Drug Administration, 2022a). The underlying mechanism for sarilumab-induced neutropenia seems to be the same as for tocilizumab. A decreased neutrophil count is observed within hours of the administration of both drugs. The hypothesis is that IL-6 promotes demargination of neutrophils, as was elucidated in rabbits who developed neutrophilia following administration of recombinant IL-6. Thus, the administration of IL-6 inhibitors would result in margination of neutrophils and, therefore, neutropenia. A recent population pharmacodynamic model developed to assess the changes in absolute neutrophil count in blood following administration of subcutaneous sarilumab supports this hypothesis (Kovalenko et al., 2020). No immune-related mechanism has been attributed to sarilumab-induced neutropenia. Thrombocytopenia of fewer than 100,000 per mm^3^ occurs in approximately 1% of patients receiving sarilumab (Fleischmann et al., 2020). The mechanism whereby sarilumab induces thrombocytopenia is unknown, but the hypothesis points to the same mechanism as for tocilizumab (see section 2.1.2.2). To our knowledge, no immune-related mechanism has been linked to sarilumab-induced thrombocytopenia.

##### 2.2.2.3 Recommendations

The use of sarilumab is not recommended during the course of an active infection. Laboratory parameters that should be monitored are neutrophil count, platelet count, liver enzymes, and lipid profile. The drug should not be initiated in patients with an absolute neutrophil count of fewer than 2,000/mm^3^, platelets less than 150,000/mm^3^, or if liver enzymes are 1.5 times the upper limit of normal (Food and Drug Administration, 2022a).

### 2.3 Siltuximab

Siltuximab is a chimeric monoclonal antibody that forms high-affinity complexes with human IL-6. It is used primarily to treat multicentric Castleman’s disease (MCD) (Food and Drug Administration, 2022b). Currently, there is no recommendation for the use of siltuximab in COVID-19. There are few studies evaluating the efficacy and safety of this drug in COVID-19; however, due to the mechanism of action, it is being considered as a possible candidate. Observational cohort studies showed that patients with severe COVID-19 requiring ventilatory support might benefit from treatment with siltuximab to reduce mortality (Gritti and Giovanni, 2022). However, in a RCT that evaluated the role of IL-1 and IL-6 blockade on time to clinical improvement, siltuximab, tocilizumab, and anakinra did not show improvement (Declercq et al., 2021). Nevertheless, ongoing clinical trials are evaluating the efficacy and safety of this drug in COVID-19 (NCT04486521), (NCT04329650) (Martinez, 2022; Anti-IL6 and Corticosteroid Monotherapy, 2022). Given the future potential role of this drug in COVID-19 treatment, we decided to include it in our revision, reviewing safety data and possible irAEs.

#### 2.3.1 Safety

The most common adverse reactions in patients treated with siltuximab for MCD are pruritus, increased weight, rash, hyperuricemia, and upper respiratory tract infection (Food and Drug Administration, 2022b). Nevertheless, in another study, a long-term assessment of the safety of siltuximab revealed that it is generally well-tolerated, some patients presented hypertension, fatigue, nausea, neutropenia, vomiting, and infection, while some presented serious adverse events including polycythemia and urinary retention (van Rhee et al., 2020). Furthermore, in a systematic review of patients with MCD treated with siltuximab the reported adverse events were hypertension, nausea, fatigue, cellulitis, hypertriglyceridemia, and hypercholesterolemia, while serious adverse effects were leukopenia, lymphopenia, and polycythemia (Rhee et al., 2015; Sitenga et al., 2018).

#### 2.3.2 Immune-related adverse drug reactions

##### 2.3.2.1 Hypersensitivity reactions and immunogenicity

Hypersensitivity reactions with siltuximab are expected to be higher than those encountered with tocilizumab or sarilumab, given that this is a chimeric antibody and not a humanized or fully human antibody (Table 2). Hypersensitivity reactions are reported in approximately 4.8% of patients, of which 0.8% were considered to be severe (Chmp, 2021). Furthermore, among the most frequently reported adverse reactions besides infections were pruritus and a maculopapular rash, both of which may represent an IgE-mediated hypersensitivity reaction. Regarding the potential immunogenicity of siltuximab, anti-drug antibodies were tested in 411 patients, and only one patient (0.2%) resulted positive, with no neutralizing capability and low titer.

**TABLE 2:** Incidence of hypersensitivity reactions with the use of different IL-6 inhibitors.

Drug	% Of Human Homology	Incidence of hypersensitivity reactions	Reference
Tocilizumab	90% (humanized monoclonal antibody)	IV: 0.1% SC: 0.7%	Actemera, (2022)
Sarilumab	99% (fully human antibody)	0.3%	Food and Drug Administration, (2022a)
Siltuximab	65% (chimeric antibody)	4.8%	Chmp, (2021)

IV, intravenous, SC, subcutaneous.

##### 2.3.2.2 Hematologic Abnormalities

Neutropenia, thrombocytopenia, and lymphopenia have been reported in some cases with the use of siltuximab (Rhee et al., 2015; Sitenga et al., 2018). There are no studies pointing towards an immune-mediated mechanism for these hematologic abnormalities; in the case of neutropenia and thrombocytopenia, we believe the same mechanism seen in tocilizumab would apply to this drug (see section 2.1.2.2). Lymphopenia has been documented in the long-term evaluation of siltuximab (Rhee et al., 2015), with a mean treatment duration of 5.1 years, and was present in one out of 19 patients. Although this association is rare, there are hypotheses as to why IL-6 inhibitors could potentially cause lymphopenia. They principally point out that IL-6 mediates the anti-apoptotic function of mesenchymal stem cells on lymphocytes. Thus, inhibiting IL-6 could lead to increased lymphocyte apoptosis (Xu et al., 2007).

##### 2.3.2.3 Recommendations

From a biological point of view, the use of siltuximab has more risk of presenting hypersensitivity reactions than the other IL-6 antagonists because it is a chimeric monoclonal antibody, so the clinicians should be aware of any signs pointing to a hypersensitivity reaction. Infusion of siltuximab should be stopped if the patient develops signs of anaphylaxis. The infusion should also be stopped if there are mild to moderate infusion reactions. The patient’s absolute neutrophil count, platelet count, and hemoglobin should be measured before the first administration of siltuximab. The neutrophil count should be ≥ 1.0 × 10^9^/L, platelets ≥75 × 10^9^/L, and hemoglobin ≤17 g/dl (Food and Drug Administration, 2022b).

## 3 Anti-SARS-CoV-2 monoclonal antibodies

### 3.1 Sotrovimab

Sotrovimab is a human monoclonal antibody that binds to the receptor-binding domain of the spike protein of SARS CoV-2, on an epitope that is highly conserved among multiple other sarbecoviruses (Annex, 2021). This prevents viral attachment and posterior infection to the host cell. The use of sotrovimab has been approved by regulatory agencies for emergency use for the treatment of mild to moderate COVID-19 in non-hospitalized patients (Emergency Preparedness And Response Mcm Legal, 2022) due to the interim results of a clinical trial that showed a reduced risk of disease progression among high-risk patients with mild to moderate COVID-19 (Gupta et al., 2021). Thus, it has been approved for patients of 12 years of age or more with a positive SARS CoV-2 test who have a high risk for disease progression (older age, obesity, diabetes, chronic lung diseases). Nevertheless, concerns have been raised about the efficacy of this drug against SARS CoV-2 variants with mutations on the spike protein (Das et al., 2021; Das et al., 2022). Indeed, as of april 5, 2022, the FDA revoked the emergency use authorizations in any U.S. (Unites States) region due to the increase of COVID-19 cases caused by the sub-variant omicron (FDA, 2022).

#### 3.1.1 Safety

The majority of information regarding the safety of sotrovimab in COVID-19 is based on the COMET-ICE trial. This clinical trial included 1,057 non-hospitalized participants with mild to moderate COVID-19 who have at least one risk factor for progression, randomized to receive intravenous sotrovimab or placebo. This trial showed that all-cause hospitalization lasting longer than 24 h or death was reduced with the use of sotrovimab. Regarding safety, adverse events were reported in 22% of patients in the sotrovimab group; the most common was diarrhea, followed by nausea and headache. Systemic infusion-related reactions, including rash and pruritus, were reported in six patients in the sotrovimab group (Gupta et al., 2022b). Cases of anaphylaxis following infusion of sotrovimab have been described in other studies (Sotrovimab Product Monograph, 2022). There have been no reports of abnormal laboratory findings, nor other post-market adverse reactions.

#### 3.1.2 Immune-related adverse drug reactions

##### 3.1.2.1 Hypersensitivity reactions and immunogenicity

Immediate hypersensitivity events following treatment with sotrovimab are estimated to occur in 1% of treated patients (Gupta et al., 2022b). Infusion-related reactions have also been observed including fever, difficulty breathing, chills, fatigue, chest pain, altered mental status, bronchospasm, angioedema, rash, pruritus, myalgia, and diaphoresis. Anti-sotrovimab antibodies have been found in preclinical studies (CHMP, 2021); however their effect on the safety or efficacy of sotrovimab is not known due to limited data.

##### 3.1.2.2 Recommendations

Currently, there are no reports of laboratory abnormalities, and available recommendations are directed towards infusion-related reactions. The infusion rate is recommended to be slowed or stopped if the patient develops an infusion-related reaction, defined as any event happening until 24 h after the injection. In addition, infusion of the drug should be made in a controlled environment with the capacity to respond in case of anaphylaxis, and the patient should be monitored during and 1 h after drug administration.

## 4 Alternative treatment options

Despite the low documented incidence of side effects associated with these immunotherapies and the low likelihood of presenting a severe adverse reaction, some patients may experience serious adverse effects that warrant treatment discontinuation. In those cases, it is relevant to know what other therapies are available for the treatment of COVID-19 induced CSS. Patients that develop CSS are found to have increased levels of IL-6, but also IL-1β (Liu et al., 2020). Consequently, other biological immunotherapies that target IL-1 are available and are reasonable therapeutic alternatives for patients that present hypersensitivity reactions to drugs targeting IL-6 (siltuximab, sarilumab, tocilizumab). For instance, Marc et al. evaluated the use of remdesivir plus tocilizumab compared to remdesivir plus anakinra (an IL-1 receptor antagonist) in patients with moderate to severe COVID-19 pneumonia. There was clinical and paraclinical improvement in both groups, with a decrease in the parameters of cytokine storm. However, the group of tocilizumab had a much faster beneficial effect than anakinra. Nonetheless, anakinra could be a possible alternative treatment option in patients with moderate to severe COVID-19 who present serious adverse reactions to IL-6 pathway inhibitors, especially those that are in critical states (Hecker et al., 2022).

When comparing IL-1 and IL-6 inhibitors in patients with COVID-19 the group of Cavalli et al. carried out a cohort study including patients with COVID-19 with respiratory insufficiency and hyperinflammation (CRP >100 mg/L, or ferritin >900 ng/ml) (Cavalli et al., 2021). Clinical outcomes were compared for patients receiving IL-1 inhibition with anakinra or IL-6 inhibition with tocilizumab or sarilumab compared with standard of care (patients who did not receive interleukin inhibitors). Patients treated with anakinra had a significantly reduced mortality risk compared to patients who received standard of care. Il-6 inhibitors significantly reduced risk of mortality in patients who had higher levels of CRP (Cavalli et al., 2021). While targeting IL-1 seems a good alternative treatment option, other drugs targeting IL-1β such as canakinumab have shown conflicting results. In a phase III RCT canakinumab failed to demonstrate increased survival in hospitalized patients with severe COVID-19 compared to placebo (Caricchio et al., 2021). However in some observational prospective cohort studies patients treated with canakinumab had increased survival and better clinical outcomes (Landi et al., 2020; Katia et al., 2021). Anakinra is currently recommended by The National Institute of Health’s COVID-19 guidelines for the treatment of hospitalized pediatric patients with refractory multisystem inflammatory syndrome, however it is important to note that anakinra is not currently approved by the FDA for the management of COVID-19 in adult patients and that there is currently insufficient evidence to recommend for or against it (National Institutes of Health, 2022).

Itolizumab is an anti-CD6 humanized monoclonal antibody used for the treatment of chronic plaque psoriasis. It acts by preventing the binding of CD6 to ALCAM (activated leukocyte cell adhesion molecule) which in turn inhibits T cell activation, proliferation, differentiation, and survival (Gore and Kshirsagar, 2021). Itolizumab subsequently reduces the production of pro-inflammatory cytokines by blocking the activation and trafficking of T effector cells (Loganathan et al., 1025). Given its mechanism of action, treatment of COVID-19-induced CSS with itolizumab has been investigated. In a phase II RCT itolizumab-treated patients had a reduction in 1-month mortality rate and improved SpO2, PO2, decreased levels of IL-6 and TNF-α. Furthermore, the reported adverse reactions were transient lymphopenia and infusion reactions, with no report of serious adverse effects (Kumar et al., 1080). Furthermore, in an observational cohort study, patients treated with itolizumab showed a significant decline of IL-6, C reactive protein and ferritin, increased oxygen saturation and accelerated recovery in adult patients (Gore and Kshirsagar, 2021). However, itolizumab is not currently recommended for the treatment of COVID-19 and more information is needed to recommend its use.

Apart from biological immunotherapies, other alternative treatment options include JAK (Janus kinase) inhibitors such as baricitinib. JAKs are tyrosine protein kinases that modulate signals from cytokines by activating Signal Transducers and Activators of Transcription (STATs) modulating gene transcription of inflammatory mediators. Baricitinib is currently approved by FDA under emergency use authorization, in patients with COVID-19 requiring supplemental oxygen, invasive or non-invasive ventilation or ECMO (Food and Drug Administration, 2022c). Baricitinib is a reasonable alternative to tocilizumab. It is recommended in hospitalized patients with COVID-19 that require oxygen through a high-flow device, receiving dexamethasone with rapidly increasing oxygen needs and systemic inflammation (National Institutes of Health, 2022). The efficacy of baricitinib was evaluated in a systematic review and meta-analysis that included clinical trials and observational studies. The use of baricitinib was found to be associated with a reduction in mortality in patients with COVID-19, reduced risk of ICU admission, reduced the need for invasive mechanical ventilation and showed no differences in safety compared to the control group (Lin et al., 2022). Thus, we consider that patients with severe COVID-19 that present serious adverse reactions with IL-6 pathway inhibitors should be managed with baricitinib as an alternative treatment option to manage the excessive immune response seen in these patients.

## 5 Conclusion

In conclusion, the use of biological immunotherapies in COVID-19 has shown promising results. Some of them have shown improved clinical outcomes principally through the modulation of the excessive immune response seen in severe cases of COVID-19. However, there are still many studies missing before we can completely determine the safety of these drugs in COVID-19. In general, all the reviewed drugs had documented cases of anaphylaxis and other hypersensitivity reactions. However, risk factors for the occurrence of these are largely unknown. Therefore, there is a need to identify the associated factors that could contribute to hypersensitivity reactions, given the severity of some of them. Furthermore, besides hypersensitivity reactions, only lung injury induced by tocilizumab was linked to a possible immune-related response, hypothesized to be secondary to immune complex deposition. In addition, mechanisms for most hematological abnormalities seen with the use of tocilizumab, sarilumab, and siltuximab are not known. Studies clarifying this mechanism would be of great utility, especially in cases where the treatment needs to be stopped due to severe neutropenia/thrombocytopenia, and where treatment or prevention of this adverse drug reaction would be beneficial.
